# All That Glitters in cfDNA Analysis Is Not Gold or Its Utility Is Completely Established Due to Graft Damage: A Critical Review in the Field of Transplantation

**DOI:** 10.3390/diagnostics13121982

**Published:** 2023-06-06

**Authors:** Victor Jiménez-Coll, Jaouad El kaaoui El band, Santiago Llorente, Rosana González-López, Marina Fernández-González, Helios Martínez-Banaclocha, José Antonio Galián, Carmen Botella, María Rosa Moya-Quiles, Alfredo Minguela, Isabel Legaz, Manuel Muro

**Affiliations:** 1 Immunology Service, University Clinical Hospital Virgen de la Arrixaca, Biomedical Research Institute of Murcia (IMIB), 30120 Murcia, Spain; 2 Nephrology Service, University Clinical Hospital Virgen de la Arrixaca, Biomedical Research Institute of Murcia (IMIB), 30120 Murcia, Spain; 3 Department of Legal and Forensic Medicine, Biomedical Research Institute of Murcia (IMIB), Faculty of Medicine, Regional Campus of International Excellence “Campus Mare Nostrum”, University of Murcia, 30100 Murcia, Spain

**Keywords:** donor-derived cell-free DNA (cfDNA), graft injury, acute rejection, organ transplant, monitoring

## Abstract

In kidney transplantation, a biopsy is currently the gold standard for monitoring the transplanted organ. However, this is far from an ideal screening method given its invasive nature and the discomfort it can cause the patient. Large-scale studies in renal transplantation show that approximately 1% of biopsies generate major complications, with a risk of macroscopic hematuria greater than 3.5%. It would not be until 2011 that a method to detect donor-derived cell-free DNA (dd-cfDNA) employing digital PCR was devised based on analyzing the differences in SNPs between the donor and recipient. In addition, since the initial validation studies were carried out at the specific moments in which rejection was suspected, there is still not a good understanding of how dd-cfDNA levels naturally evolve post-transplant. In addition, various factors, both in the recipient and the donor, can influence dd-cfDNA levels and cause increases in the levels of dd-cfDNA themselves without suspicion of rejection. All that glitters in this technology is not gold; therefore, in this article, we discuss the current state of clinical studies, the benefits, and disadvantages.

## 1. Introduction

In kidney transplantation, a biopsy is currently the gold standard for monitoring the transplanted organ. A biopsy involves taking a small tissue sample from the transplanted kidney, which is then examined under a microscope to assess organ health. This is done to assess for any signs of rejection or other problems that may be occurring in the transplanted kidney. However, this is far from an ideal screening method given its invasive nature and the discomfort it can cause the patient. Large-scale studies in renal transplantation show that approximately 1% of biopsies generate major complications, with a risk of macroscopic hematuria greater than 3.5% [1]. Most biopsy-related complications, such as pain and bleeding, are minor and localized, and can be managed conservatively [2]. The most severe complication, however, is the risk of perforation of the collecting system or the kidney itself, which can result in severe hemorrhage, sepsis, and even death. To minimize the risk of complications, careful patient selection, proper imaging guidance, and specialized instruments and techniques are essential [3,4]. Imaging techniques, such as ultrasound and computed tomography (CT), are essential for accurate needle placement. Ultrasound imaging is the most commonly used modality for needle guidance due to its versatility, cost-effectiveness, and relative safety. It allows for real-time visualization of the renal transplant and the surrounding anatomy, making it ideal for guiding percutaneous needle placement. CT imaging can also be used but is typically reserved for more complex cases with insufficient ultrasound imaging.

In addition, with current immunosuppressive therapies, the detection of subclinical rejection is too infrequent to justify this risk, which has meant that many units no longer perform these routine biopsies [5], thus raising the urgent need to find a new non-invasive biomarker that allows the detection of said rejection in order to intervene in time or modify immunosuppression. In response to this need, recent studies have shown that non-invasive biomarkers could be a viable option for detecting subclinical rejection [6,7,8,9]. These non-invasive biomarkers include urinary and serum markers such as urinary albumin-to-creatinine ratio (UACR) and donor-specific antibodies (DSA). Additionally, imaging techniques, such as magnetic resonance imaging (MRI) and ultrasound (US), have been used to detect graft changes that may signal rejection [10,11,12]. Finally, genetic and epigenetic biomarkers, such as microRNAs, have also been used to detect subclinical rejection [13,14,15]. Ultimately, using these non-invasive biomarkers could help identify and intervene in cases of subclinical rejection earlier, thus avoiding more serious complications.

Currently, serum creatinine and urinary indicators are the ones that fulfill this role because they are cheap, easily interpretable, and relatively reliable [16]. The creatinine level in the blood measures kidney function, and an increased creatinine level indicates a decrease in kidney function [17]. Urinary indicators, such as the urine protein/creatinine ratio, are used to assess kidney function [18]. These tests can indicate the health of the kidneys. Other tests, such as blood urea nitrogen and glomerular filtration rate, are also used to assess kidney function [19]. However, these tests are more expensive and require specialized equipment. They are also more challenging to interpret and can be affected by factors such as hydration status.

In conclusion, serum creatinine and urinary indicators are the most commonly used tests for assessing kidney function due to their affordability, ease of interpretation, and reliability. However, its sensitivity and specificity in detecting allograft damage are poor. The recent approval of tests to detect donor-derived cell-free DNA (dd-cfDNA) has given great hope in the non-invasive recognition of allogeneic damage.

## 2. Types de Cell-Free DNA

Degraded DNA fragments released into the blood or other fluids are known as cfDNA. Its first detection dates back to 1948 in patients with systemic lupus erythematosus [20]. It would not be until 1970 that this new biomarker would begin to be considered helpful for the clinic, as researchers observed differences in its concentration depending on the health status of the individual studied and began to see its application in cancer patients by allowing the detection of fragments of tumor DNA in the blood [21]. Its interest increased when it was discovered that tumor cells not only released cfDNA into the bloodstream but that these fragments also had the genetic and epigenetic changes of the tumor cells from which they had originated [22]. Shortly after, analysis of fetal cfDNA in maternal plasma began to be used to detect Rh mismatches and chromosomal aneuploidies [23].

Recent literature shows that different cfDNA types can be used as biomarkers of various disease states [16,24]. The following stand out for their relevance: ccf mtDNA (circulating cell-free mitochondrial DNA), ctDNA (circulating tumor DNA), cffDNA (cell-free fetal DNA), and dd-cfDNA (donor-derived cell-free DNA). These types and their applications are listed in Table 1.

## 3. History of Donor-Derived Cell-Free DNA

Non-patient cfDNA from a transplanted organ is known as circulating dd-cfDNA and can be detected in both blood and urine. Initially, the dd-cfDNA concentration increases to values greater than 5% after transplantation; however, these decrease rapidly and are practically undetectable after a week. Therefore, the presence of dd-cfDNA in the blood of the transplanted patient after a short period may be related to possible complications [1,26]. If the transplanted individual rejects the graft, the concentration of dd-cfDNA increases up to five times more than in healthy controls. In addition, these increased levels can be identified before any other clinical or biochemical symptom or complication, which is why it is of great interest for the early approach to subclinical rejection [27].

In 1998, this interesting biomarker was detected for the first time in the plasma and urine of solid organ recipients, identifying DNA from the Y chromosome in women transplanted with organs from male donors [27,28]. After this discovery, the technology began to evolve, emerging quantitative PCR techniques oriented to typing HLA genes. However, they had reproducibility problems and could not distinguish donor and recipient if they shared typings [5,29].

It would not be until 2011 that a method to detect dd-cfDNA employing digital PCR was devised based on analyzing the differences in SNPs between donor and recipient [30]. This strategy was optimized in 2016 with other authors [31] who, based on the principles of allelic imbalance, were able to measure dd-cfDNA levels by genotyping only the recipientm and between 150,000 and 600,000 SNPs from the donor, therefore, not being necessary to genotype the latter [15] altogether. Later that same year, other authors demonstrated that it was possible to quantify cfDNA levels by analyzing only 266 carefully chosen SNPs to minimize the probability that two unrelated individuals share them [32]. However, although these discoveries have laid the foundations for commercial dd-cfDNA detection kits, they have a series of limitations that have not yet been resolved, such as the impossibility of detecting dd-cfDNA levels in the transplantation of identical twins or differentiating the presence of more than two different genomes, as occurs in the case of retransplanted patients [16,33].

## 4. Commercial Tests for dd-cfDNA Detection

Three commercial dd-cfDNA detection kits are available for clinical use in kidney transplantation; Allosure from CareDx, Prospera from Natera, and Trac from Viracor Eurofins [25,29,34]. The most commonly used and described in various publications is CareDX, where a panel of 266 SNPs in 22 somatic chromosomes was used to study 102 kidney transplant recipients, 27 with rejection confirmed by biopsy [33]. In the said test, a 1% dd-cfDNA was established as a cut-off to discriminate the presence or absence of active rejection, and it had a specificity of 85%, a sensitivity of 59%, a positive predictive value (PPV) of 61% and an 84% of accuracy negative predictive value (NPV). In this study, TCMR was insufficiently detected, as confirmed by Huang in an independent study with the same test [35]. One reason may be using relatively long amplicons (100–130 bp) in the employed test. According to Clausen et al. [36], the recommended amplicon length is 85.4 bp (66–103 bp).

Prospera de Natera’s dd-cfDNA levels were determined in the following study by detecting 13,392 SNPs on four chromosomes [30,37], and a retrospective study was performed on 178 kidney transplant patients diagnosed with rejection. Acute T-lymphocyte-mediated (TCMR) or antibody-mediated (ABMR). Based on the results obtained, a cut-off for the presence of rejection more significant than 1% of dd-cfDNA was established, as in the Allosure method, with a specificity of 73%, a sensitivity of 89%, a PPV of 52%, and an NPV of 95% [38]. TCMR was well detected, presumably due to shorter amplicon size. Similar results were obtained in the Trifecta study [39].

These two studies would be the most relevant to date at the level of bibliographic background; however, a large number of companies are trying to develop dd-cfDNA kits, and that is where the Eurofins TRAC (Transplant Rejection Allograft Check) study would come in, which uses NGS techniques and recipient genotype data to determine the percentage of dd-cfDNA from the donated organ. In said study, biomarkers were determined in 77 kidney transplant patients with a cut-off of 0.70% to discriminate active rejection and sensitivity values of 58%, specificity of 85%, PPV of 55%, and NPV of 86% (Eurofins study) [38].

**Figure 1:**
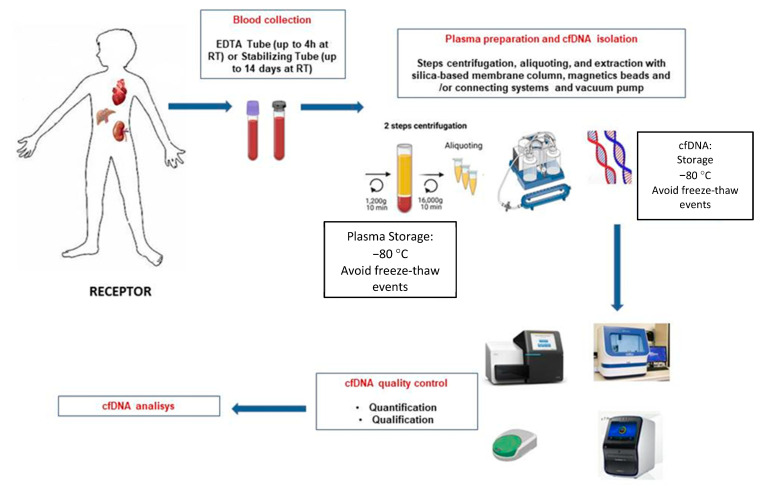
Schematic overview of the different steps for blood sample processing of graft recipients and cfDNA extraction.

To clarify and inform the particular process, a schematic illustration of cfDNA isolation and analysis is shown in Figure 1.

These commercial kits for determining dd-cfDNA represent a significant advance in the early detection of subclinical rejection; however, their main drawback is that their prohibitive price means that they are still not valid as a detection technique screening [33,40]. Another group carried out an economic analysis in which commercial dd-cfDNA tests were used as screening for subclinical rejection in kidney transplant patients and concluded that this system was not cost-efficient and that, in order to reach so, the price of each test should be less than US$700, instead of the US$2200–2800 it currently ranges from [38]. That is why new technologies aimed at minimizing this cost are beginning to emerge, such as those based on dd-PCR [41], which reduce the price to less than US$400. However, this technology is relatively recent and has few studies that endorse it [42].

## 5. Current Status of dd-cfDNA

Leaving economic aspects aside, dd-cfDNA has the problem of having a relatively low PPV (although it should be mentioned that predictive values also depend on the variable prevalence of rejection in the study populations), which makes it difficult to interpret a high level of dd-cfDNA in the absence of additional clinical information, which is why it is often used, together with the analysis of leukocyte expression markers. However, its NPV is much higher, which allows it to play an essential role in avoiding unnecessary biopsies [43]. In addition, it is important to remember that other types of pathologies, independent of rejection, can raise dd-cfDNA levels, so it is essential to evaluate the results within the patient’s clinical context [33,44,45,46,47,48]. The absolute quantification of dd-cfDNA (cp/mL) has the advantage of not being affected by changes in the cfDNA receptor [49,50]. Leukopenia and leukocytosis can alter the dd-cfDNA fraction and produce false positive or negative results [41,51,52]. An example of these facts was the studies carried out by another group in 2020, which retrospectively studied elevations in dd-cfDNA levels in patients with BK viremia; however, these studies did not reach statistical significance [53]. Another group worked along the same lines, observing a positive correlation between BK viremia and increased dd-cfDNA levels in a small cohort of 10 patients [54].

In addition, since the initial validation studies were carried out at the specific moments in which rejection was suspected, there is still not a good understanding of how dd-cfDNA levels naturally evolve post-transplant. In addition, various factors both in the recipient (such as the panel reactive antibody [PRA]) and in the donor (living transplant or after circulatory death [DCD]) can influence dd-cfDNA levels and cause increases in the levels of dd-cfDNA themselves in the absence of suspicion of rejection [33]. Another physiological factor influencing dd-cfDNA that is being studied is obesity, with a retrospective study (in 2020) showing an inverse relationship between morbid obesity and dd-cfDNA levels [55].

The predictive value of dd-cfDNA has been extensively analyzed in acute cellular rejection (ACR) cases and AMR, the two main mechanisms of allograft damage. Among the multiple studies carried out, the most relevant was the so-called DART (Diagnosing Acute Rejection in Kidney Transplant), carried out in the United States with a representative sample of the kidney transplant population (*n* = 384) [37], which reports a better predictor of AMR than of ACR, since the levels of the biomarker in the first case are much higher, ranging between 1.4% and 2.9%. In comparison, for ACR, they are around 1.2%.

## 6. Active Studies

Given the great potential of dd-cfDNA, many active clinical trials are delving into it, both by commercial houses to improve their products and by hospitals and university entities seeking to expand knowledge and technology, and the clinical utility of this new biomarker is shown in Table 2.

The different methods of cfDNA analysis have also advantages and limitations. For example, in the case of PCR-based methods, these analyses could be performed by qPCR and digital PCR. In the first case of qPCR, high specificity and sensitivity, cost-effectiveness, rapid, and ease of use are essential characteristics, and its limitations are no multiplexing and limited to detecting known mutations. In the second case of digital PCR, the particular advantages would be up to five targets, high sensitivity and specificity, absolute quantification, single molecule analysis, cost-effective, rapid and ease of use, and its limitations are limited multiplexing (number of fluorescent colors) and limited to detection of known mutations.

In the case of PCR coupled to spectrometry, the advantage is multiplexing capacity, and its limitation would be that this method is also limited to detecting known mutations as previous methods.

Finally, the NGS-based methods can be targeted or untargeted. In the first case of targeted, the methods or techniques [Tam-Seq, eTam-Seq, Safe-SeqS, duplex sequencing, TEC-Seq, Single primer extension (SPE), SPE-duplex UMI, CAPP-Seq, iDES eCAPP-Seq, or Ig-HTS] have advantages as high and very high specificity, error correction, error correction by SSCS (singe stranded consensus sequence), error correction by DSCS (double-stranded consensus sequence), hybrid capture method (not dependent on fragment size), amplicon methods by SPE (not dependent on fragment size), error correction by SSCS and/or error correction by DSCS and correction of stereotypical error and their limitation are amplicon methods by multiplex PCR (depend on fragment size), less comprehensive than WGS (whole genome sequencing) or WES (whole exome sequencing), need extensive input, allelic bias (capture), stereotypical errors (hybridization steps) or tissue biopsy needed.

In the case of untargeted, the advantages are mutation discovery and signatures, CNV (copy number variation), fusion genes, rearrangements, predicted neoantigens, tumor mutational burden, shallow sequencing, genome-wide, profiling, and identification of cancer signatures. The limitations would be low sensitivity (increasing depth leads to high cost), need for bioinformatics expertise, expensive, variable sensitivity, and lots of data generated.

## 7. Future Directions

In the field of kidney transplantation, a biopsy is currently the only reliable method to detect graft damage, so there is an urgent need to find a non-invasive technique or a biomarker that allows anticipating the deterioration of kidney function in order to intervene early or modify immunosuppression [56,57,58]. Currently, serum creatinine and urinary indicators are the ones that fulfill this role because they are cheap, easily interpretable, and relatively reliable. However, their sensitivity and specificity in detecting allograft damage are poor. For this reason, the recent approval of tests to detect dd-cfDNA has given great hope in the non-invasive recognition of allogeneic damage. The use of dd-cfDNA tests is growing in importance due to their sensitivity and specificity, which allow early and reliable detection of allograft damage. Additionally, dd-cfDNA tests are easier to use and interpret than conventional tests, as they do not require sample manipulation and can be performed with a single sample. Furthermore, they provide a more comprehensive range of information, such as the presence of donor-specific antibodies (DSA), which can be used to tailor treatment in the case of allograft injury.

After a kidney transplant, various causes can cause graft failure. On the one hand, a rejection of the transplanted kidney can occur. Rejection episodes can be mild, moderate, or severe. In addition, the risk of infection is increased after a transplant due to the use of immunosuppressive drugs. Infections can lead to graft failure if not treated on time.

On the other hand, if the transplanted kidney does not receive enough blood, it may not work correctly, and the graft may fail. Damage to transplanted kidney tissue can also occur from physical trauma, surgery, or certain medications. Blood clots can form in the transplanted kidney, leading to graft failure. Furthermore, using immunosuppressive drugs can cause long-term toxicity, leading to graft failure. In these cases, dd-cfDNA levels increase rapidly, which makes it possible to act in the face of subclinical rejection more quickly and efficiently than just measuring creatinine. This can allow for earlier intervention when subclinical rejection occurs, preventing further damage to the organ. In addition, because dd-cfDNA levels are a better indicator of subclinical rejection than creatinine, clinicians can be more confident in their diagnosis and treatment. In this way, identifying an early increase in dd-cfDNA levels in the receptor would not only reduce the number of unnecessary biopsies. However, it would also provide the clinician with essential information to modify the immunosuppressive therapy at the appropriate time and prevent the progression of the damage [59,60]. Identifying an early increase in dd-cfDNA levels in the receptor could reduce unnecessary biopsies and provide the clinician with essential information to modify the immunosuppressive therapy at the appropriate time and prevent the progression of the damage. This would benefit both the patient and the healthcare system, reducing costs and improving patient outcomes.

However, despite the eventual promise of this biomarker, many aspects of it and its implementation are still not fully controlled, so further investigations are necessary. As previously highlighted, it is still unclear how different donor and recipient parameters can influence this measurement and alter its results. In addition, there is still no consensus on which clinical data should prevail and whether the absolute value of dd-cfDNA or the percentage should be standardized to optimize clinical practice [8,11,16,29].

Another problem currently facing the use of dd-cfDNA as a biomarker in clinical practice is that most commercial kits require sending blood samples to an external laboratory for analysis, leading to inconsistent results. cf-DNA is quite unstable, and the transfer and transport processes and the delay in sample processing can alter the results, so it would be necessary to establish the possibility of performing these tests in the transplant center itself [33].

Lastly, another critical factor for health management is the current high economic cost of cfDNA determination, especially with NGS technology, which makes eventual implementation in a clinical service more expensive.

Therefore, despite the strong demand for a new biomarker to predict rejection and the great hope placed on dd-cfDNA, today, the number of variables that surround it means that it cannot yet reach the clinical relevance that is expected, as highlighted in two recent meta-analyses that conclude that, even though dd-cfDNA may become a very valuable marker for detecting AMR in kidney transplant patients, it is not yet fully demonstrated or established when it comes to coping with cell rejection [60,61]. This does not mean that research should be abandoned, but rather that more studies should be conducted to understand better the role that dd-cfDNA can play in predicting rejection and to develop better methods for interpreting the results obtained from the tests. The potential of longitudinal monitoring for personalized immunosuppression should be addressed [62]. Medicare provides coverage for dd-cfDNA routine testing in the US based on current evidence [35].

With further research, it is possible that dd-cfDNA could become a reliable and clinically relevant tool for predicting rejection and improving patient outcomes in kidney transplantation.

In conclusion, dd-cfDNA is a promising biomarker capable of predicting acute rejection organ transplants, but more and more studies should be performed to complete its implementation in routine clinical practice.

## Figures and Tables

**Table 1:** Different types of cfDNA and their main clinical applications.

Type of Cell-Free DNA	Abbreviations	Potential Application
Circulating cell-free mitochondrial DNA	ccf mtDNA	Diagnostic and predictive markers in various disease states, markers of cell death, and non-specific tissue damage
Circulating tumor DNA	ctDNA	Marker in oncological diagnostics, monitoring of tumor development
Cell-free fetal DNA	cffDNA	Prenatal diagnostics, detection of fetal defects
Donor-derived cell-free DNA	dd-cfDNA	Evaluation of post-transplant complications

Extracted from [25].

**Table 2:** Compilation of currently active clinical trials.

Title of Assay	Institution	Type of Study	Cohort	Criteria of Exclusion	Primary Objective	Date of the End
Study in Detection cfDNA for the Early-Stage Diagnosis of Acute Rejection Post-Renal Transplantation	Renji Hospital	Observational	60 participants (20 years to 60 years)	Failure in the transplant surgery, no urine one week after the surgery, severe infectious complications, tumors, or pregnancy	Renal allograft injury	30 September 2021
Integration of Donor-derived Cell-free DNA With HLA-DR + TNFR2 + Regulatory T Cell in the Prediction of Acute Rejection and Graft Function After Kidney Transplantation	Loma Linda University	Observational	150 adult kidney transplant candidates/recipients	Age less than 18, multi-organ transplants, kidney transplant candidates/recipients with HIV or HCV	Test whether integrating dd-cfDNA fraction with HLA-DR + TNFR2 + Tregs in advance or at the time of allograft injury can improve the predictive performance for acute rejection after kidney transplantation	1 October 2026
Trifecta-Kidney cfDNA-MMDx Study	University of Alberta	Observational	300 patients	Patients will be excluded from the study if they decline participation or cannot give informed consent or multiple organ recipients.	Determine if Prospera blood test can replace kidney biopsy test	December 2023
Allosure in Simultaneous Pancreas Kidney Transplant	Washington University School of Medicine	Observational	50 patients over 18 years old	Presence of non-renal or pancreas transplanted organ	This study will observe donor-derived cell-free DNA percentages (via the Allosure test) in combined kidney-pancreas transplant recipients to establish both stable and dysfunctional Allosure essay level	31 December 2024
Study for Detection of Donor-derived Cell-free DNA After Renal Transplantation Using Devysers NGS-based Chimerism Assay	Sheba Medical Center	Observational	50 patients over 18 years old	Patients with primary non-function or those that lost their graft during the first three months	Degree of chimerism of cf-DNA	31 March 2024
Donor-Derived Cell-Free DNA for Surveillance in Simultaneous Pancreas and Kidney Transplant Recipients	Rush University Medical Center	Observational	48 patients over 18 years old.	Recipients of other solid organ transplants apart from SPK Recipients of pancreas and kidney allografts from separate donors	Determine median AlloSure level in SPK recipients with stable allograft function	1 December 2022
Eliminating the Need for Pancreas Biopsy Using Peripheral Blood Cell-free DNA(PancDX)	University of Maryland, Baltimore	Observational	140 patients over 18 years old	Pregnant women and patients undergoing multi-organ transplants	To correlate circulating dd-cfDNA to clinical and sub-clinical acute rejection in PTA, PAK, and SPK allograft recipients	31 October 2024
Study for the Prediction of Active Rejection in Organs Using Donor-derived Cell-free DNA Detection (SPARO)	Natera, Inc	Observational	500 patients over two years old	The subject received a donor organ from an identical twin	Improve Natera’s method for determining allograft rejection status using the donor-derived cell-free DNA analysis	30 October 2028
Donor-derived Cell-free DNA for Early Diagnosis of Antibody-mediated Rejection (cfDNA-DSA)	Charite University, Berlin, Germany	Interventional (Clinical Trial)	40 patients over 18 years old	Patients with pregnancy, increasing bleeding risk or multi-organ transplantation. Biopsy-proven antibody-mediated rejection	This study aims to evaluate if repeated analysis of dd-cfDNA in patients with DSA and kidney allograft biopsy, triggered by increased levels of dd-cfDNA, can lead to early diagnosis of AMBR.	1 September 2023
Use of DNA Testing to Help Transition Kidney Transplant Recipients to Belatacept-only Immunosuppression	The University of Texas Southwestern Medical Center	Interventional (Clinical Trial)	25 patients over 18 years old	Prior or concurrent non-kidney organ transplants. Presence of BK nephropathy in the current graft	Use cfDNA information to move patients to a Belatecept-only immunosuppression	31 December 2023
Cf-DNA Assay During Treatment of Acute Rejection	University of Minnesota	Observational	15 patients over 18 years old	<1-month post-transplant >12 months post-transplant	Determine whether cell-free DNA (cf-DNA) measurement can be used as a biomarker to successfully treat acute rejection (AR) episodes after kidney transplantation.	31 January 2023
Assessing Benchmarks for Allosure and Allomap Testing in Simultaneous Kidney and Pancreas Transplant Recipients. (SPKCareDx)	Montefiore Medical Center	Observational	50 participants over 18 years old	Previous history of solid organ transplantation or pregnancy	Establishing benchmarks for AlloSure and AlloMap in SPK transplant recipients with stable allograft function	13 June 2027
Blood Biomarkers in Pediatric Kidney Transplant Recipients (Omnigraf)	University of Minnesota	Observational	30 participants under 21 years	Patients who are pregnant or cannot clearly understand the conditions of the trial	Assess whether a blood biomarker can be used to monitor the response to rejection treatment in pediatric kidney transplants.	27 May 2024
AlloSure for the Monitoring of Antibody-Mediated Processes After Kidney Transplantation (All-MAP)	University of Wisconsin, Madison	Observational	69 participants	Multi-visceral transplant Contraindication to renal biopsy	Assess the role of AlloSure Donor Derived Cell-Free DNA (dd-cfDNA) assay in monitoring three high-risk groups of kidney transplant patients.	December 2023
AlloSure Guided Immuno-Optimization for COVID-19: An Early Experience(A1-COVE)	CareDx	Observational	500 participants	Not described	The utilization of AlloSure to help guide immunosuppression management in SOT transplant recipients diagnosed with COVID-19	1 March 2021

Active clinical trials were identified from a search on www.clinicaltrials.gov on 11 January 2023, under the filters “cfDNA”, “transplant”, “transplantation”, and “transplanted” SOT, solid organ transplant.

## Data Availability

Not applicable.
